# Efficacy of hyaluronic acid, absorbable collagen sponge, and their combination in minimizing bisphosphonate-related osteonecrosis of the jaws (BRONJ) after dental extraction: a preliminary animal histomorphometric study

**DOI:** 10.1186/s40902-022-00337-7

**Published:** 2022-03-01

**Authors:** Farzin Sarkarat, Alireza Modarresi, Arefeh Riyahi, Pejman Mortazavi, Fatemeh Tabandeh, Vahid Rakhshan

**Affiliations:** 1 Oral and Maxillofacial Surgery Department; Head, Craniomaxillofacial Research Center, Dental Faculty, Tehran Medical Sciences University, Islamic Azad University, Tehran, Iran; 2 Department of Oral and Maxillofacial Surgery, Gulf Medical University, Ajman, United Arab Emirates; 3 Oral and Maxillofacial Surgery Department and Craniomaxillofacial Research Center, Dental Faculty, Tehran Medical Sciences University, Islamic Azad University, Tehran, Iran; 4 Craniomaxillofacial Research Center, Dental Faculty, Tehran Medical Sciences University, Islamic Azad University, Tehran, Iran; 5 Department of Pathology, Faculty of Veterinary Medicine, Science and Research Branch, Islamic Azad University, Tehran, Iran; 6 National Institute of Genetic Engineering and Biotechnology, Tehran, Iran; 7 Department of Dental Anatomy, Tehran Islamic Azad University of Medical Sciences, Dental Branch, Tehran, Iran

**Keywords:** Bisphosphonate-related osteonecrosis of the jaws (BRONJ), Hyaluronic acid (HA), Absorbable collagen sponge (ACS), Histomorphometric analysis, Animal study

## Abstract

**Introduction:**

There is no study on the effectiveness of hyaluronic acid (HA) placement either with or without absorbable collagen sponge (ACS) in reducing or preventing bisphosphonate-related osteonecrosis of the jaws (BRONJ). This preliminary animal study examined the efficacy of this clinically important treatment.

**Methods:**

For simulating BRONJ, zoledronic acid was administered to 40 rats for 5 weeks. Two weeks later, a right first molar was extracted from each rat. The rats were randomized into four groups of socket treatments: control (empty extraction socket) or with sockets filled with ACS, HA, or HA+ACS (*n*=4×10). After 2 weeks, 5 rats in each group were sacrificed and subjected to histopathologic and histomorphometric evaluation. Eight weeks post-surgically, the rest of rats were euthanized and histologically examined. The Kruskal-Wallis test was used to compare the four treatments at each time point (*α*=0.05).

**Results:**

Six rats were lost overall. In the second week, vascularization was higher in ACS group (*P*<0.05); osteoclast activity was not different between groups (*P*>0.05); empty lacunae were the most and fewest in control and HA+ACS groups, respectively (*P*<0.05); eosinophil infiltration was maximum in HA group (*P*<0.05); lymphocyte counts were maximum and minimum in the HA+ACS and ACS groups, respectively (*P*<0.05); the highest and lowest neutrophil counts were seen in ACS and control groups, respectively (*P*<0.05); and the extent of live bone did not differ between groups (*P*>0.05). In the eighth week, vascularization was not different in groups (*P*>0.05); the highest and lowest osteoclast activities were seen in the control and HA+ACS groups, respectively (*P*<0.05); empty lacunae were the most and fewest in control and HA+ACS, respectively (*P*<0.05); maximum and minimum numbers of eosinophils were in control and HA+ACS groups, respectively (*P*<0.05); HA and control groups exhibited the highest and lowest lymphocyte counts, respectively (*P*<0.05); the lowest and highest neutrophil counts were observed in HA+ACs and control groups, respectively (*P*<0.05); and the highest and lowest extents of the live bone were observed in HA+ACS and control groups, respectively (*P*<0.05).

**Conclusions:**

Within the limitations of this preliminary animal study, HA and especially HA+ACS seem a proper method for preventing or treating BRONJ.

## Introduction

Bisphosphonates are a major medication for reducing skeletal-related mortality in osteoporosis, Paget’s disease, and hypercalcemia caused by diseases such as multiple myeloma and breast cancer. These medications inhibit bone resorption and remodeling through reducing bone turn over and inhibition of osteoclasts [1–3]. Despite their proper efficacy, they can have a frequent and severe complication, which is bisphosphonate-related osteonecrosis of the jaws (BRONJ): Following surgeries like dental extraction or periodontal surgery or implant placement or even sometimes spontaneously, the jawbone is exposed and cannot heal within 8 weeks [4–8]. The exact mechanism is unknown, but it is suggested that the complication happens due to defects in bone remodeling and wound healing, through inhibition of osteoclasts (leading to inhibition of natural bone turn over and repair of local damages) as well as affecting local bone blood supply and ischemic alterations [9].

There are methods to prevent or reduce BRONJ; these include reducing of dental infections, removing unprotected teeth, consulting the surgeon before starting bisphosphonate therapy, and reduction of the depth of periodontal pockets. However, sometimes BRONJ happens and needs to be treated. For this purpose, numerous methods have been suggested which consist of lessening BRONJ symptoms by reducing swelling and pain or purulent drainage, treating postoperative side effects (e.g., infections and dehiscences), plus debridement surgery and adjuvant conservative treatments for decreasing the number of bacteria before surgery [6, 10]. If surgery is not accepted by the patient or contraindicated (because of poor general health of the patient), the alternative remedies might be performed [6, 8].

Hyaluronic acid (HA) is a part of the extracellular matrix in most tissues of the body such as synovial fluid, connective tissue, fetal mesenchyme, skin, and many other organs and tissues of the body and as a key component in soft tissue repair. It plays a key role in bone regeneration by stimulating cell migration, adhesion, and proliferation of undifferentiated mesenchymal cells to stimulate their differentiation into osteoblast cells. It can also maintain osteoinductive factors in a localized environment due to its physical and chemical properties. In addition, it is indirectly involved in the adhesion of osteoclasts to the bone surface and can accelerate angiogenesis and bone formation [11]. In dentistry, HA can be used to treat trismus, swelling, and pain caused by extraction of the third molars, to guide tissue repair, to reduce the depth of the probe and the lesions on the radiograph, and many other cases [12]. Exogenous hyaluronic acid increases hyaluronic acid within cells as well as increases glycoprotein synthesis and decreases the formation and function of inflammatory mediators, matrix metalloproteinases, and behavioral changes in immune cells by preventing the formation of immune complex and polymorphonuclear cells, inhibiting the migration of leukocytes and macrophages, and regulating fibroblast proliferation. Many of the activities of hyaluronic acid depend on its molecular weight [13]. HA can be absorbed in the low molecular weight form and can interact with many cells (involved in the proliferation process as well as with immune cells) such as dendritic cells, macrophages, and osteoclasts [14]. Its role in the formation and strengthening of osteoclasts is also clear [15]. Therefore, it is suggested to be used as a biomaterial to enhance bone healing [11, 16–20]. However, to the best of our knowledge, no study has evaluated its effects on BRONJ.

Another material that has been suggested for enhancement of bone healing is absorbable collagen sponge (ACS). The collagen matrix is a representative organic polymer that allows stabilized release of other bone healing materials into the lesion and thus is used for periodontal and bone regeneration, usually in combination with other grafting materials [21–23]. Again, there is no study on the healing efficacy of ACS in BRONJ.

Due to the importance of BRONJ treatment and prevention as well as the lack of any studies on the efficacy of HA with or without ACS in BRONJ treatment/prevention, this animal experimental study was conducted.

## Materials and methods

This interventional prospective animal study was conducted on 40 healthy male Wistar rats; inclusion criteria were being about 220–240 g and aged 2 months old. Exclusion criteria were complications during surgery, including heavy bleeding and fractures of the jaw, trauma to rats, infections in sites other than the surgical site, intense weight loss, and rat mortality. Matching was done between groups in order to control age and gender. The study was evaluated by two different research committees and was given ethical approval by both (ethics codes: IRCT37054, IR.IAU.DENTAL.REC.1398.020). Standard plates of food and water were fed to rats, and there was no limitation on the amount and type of food intake of the rats, except in the first week after the surgery when the rats were fed soft foods to prevent potential damage from chewing on hard foods.

### BRONJ model

To establish a rat model of BRONJ, zoledronic acid was injected intravenously: After ensuring the health of the rats, zoledronic acid (Exir, Tehran, Iran) was diluted with 0.9% normal saline to a concentration of 0.2 mg/ml. Afterwards, 0.2 ml of solution once a week, which contains 0.04 mg of zoledronic acid was injected intravenously into the tails of the rats that had been disinfected with cotton wool immersed in alcohol before injection. This was done once a week, for 5 weeks.

### Right first molar extraction

**Fig. 1:**
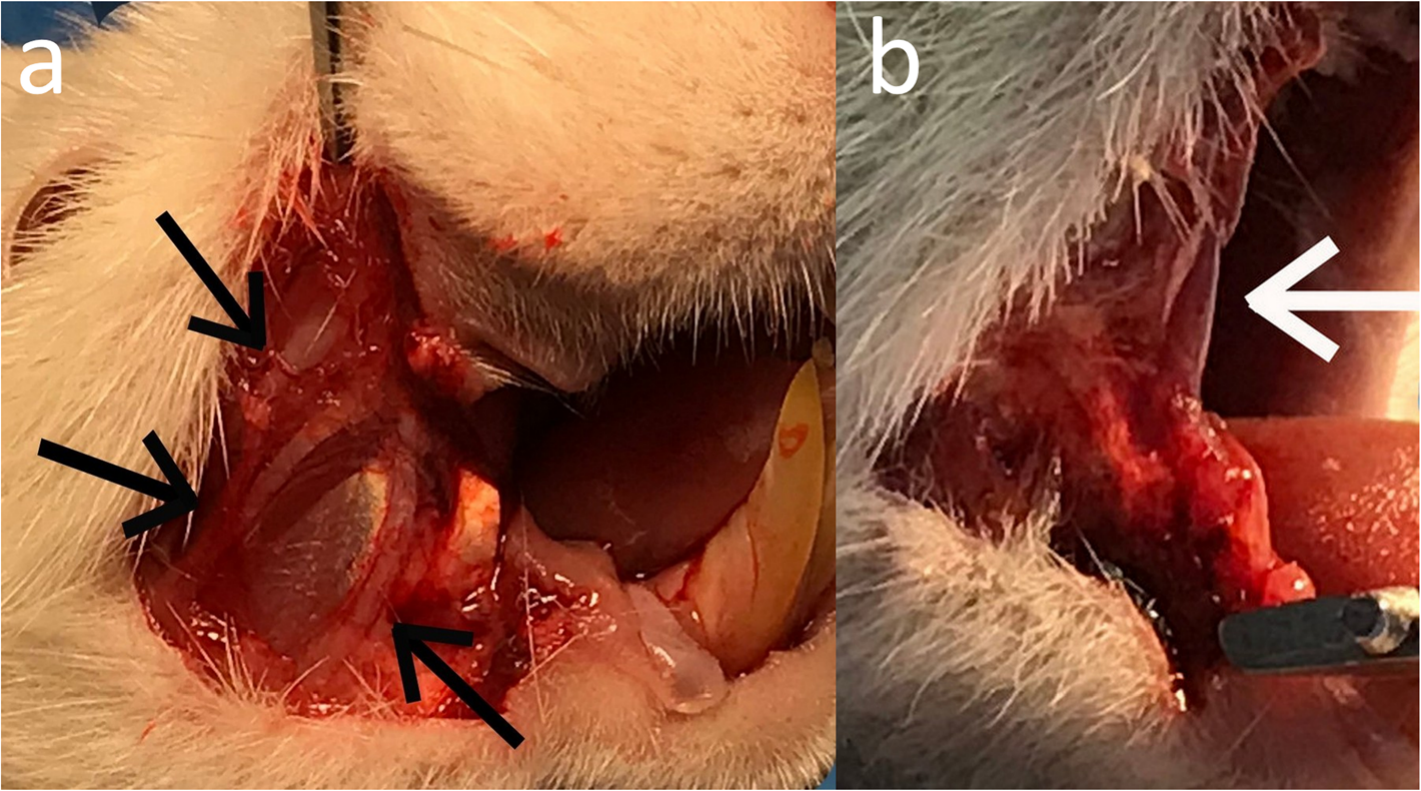
Unilateral commissurotomy is performed to increase accessibility of oral cavity. **A** The facial nerve preservation. **B** The facial artery and vein preservation

Two weeks after the completion of injections, the rat was subjected to general anesthesia induced by a mixture of ketamine 10% (100 mg/kg) and xylazine 2% (5 mg/kg) injected intraperitoneally. Their faces were scrubbed with betadine and then Commisuroplasty (comissurolusis and commissurotomy) was done at the right commissure of rats with a length of one centimeter in a plan parallel to the occlusal plane from anterior to posterior up to the anterior border of the masseter muscle. First dissection was performed in the subcutaneous area. After entering the submucosa and mucosa, they were cut to the anterior border of the masseter muscle so that the facial nerve and facial vessels were preserved (Fig. 1). Afterwards, retraction sutures were performed to increase vision and access. The rats’ mouths were opened from the opposite side (the left side) with a mouth opener and their mandibles were placed on a step for vertical support. The first right molar teeth were luxated from the mesial part by the periotome and then removed by forceps without injury or trauma.

### BRONJ treatments

The rats were randomized into four groups: three experimental groups and one control (no treatment). The control group received no treatments for BRONJ. The other groups received experimental treatments for BRONJ: Group 1, hyaluronic acid (prepared at the Tehran Genetics Research Institute with an approximate molecular weight of 400 Kda, Tehran, Iran) was applied to the socket. Group 2, ACS (Collacone, Diopars, Tehran, Iran) was applied to the socket. And in Group 3, hyaluronic acid + ACS were applied to the socket. All sockets in all 40 rats were sutured with 5/0 non-absorbable silk threads. Then the commisuroplasty areas were sutured with 4/0 nylon thread with three stitches. All surgeries were performed by a maxillofacial surgeon.

### Histological and histomorphometric examinations

At the end of the second week post-surgery, 5 rats in each group were randomly selected and euthanized by inhalation of ether solution. At the end of the eighth week, the remaining specimens were sacrificed. Then the mandible was separated from the cranial base and the entire mandible with surrounding soft tissue was fixed in 10% neutral buffered formalin (NBF, PH. 7.26). After fixation, the samples were placed in acid to be decalcified in a 3- to 4-day process. After decalcification, the desired piece was harvested using a surgical blade #20 from the mesial of the second molar to the mesial side of the extracted tooth and in the mediolateral dimension with a distance of 2 mm from the edge of the socket. Afterwards, all specimens were stored in paraffin blocks and then the tissue was cut from the middle of the mediolateral dimension to 5-μm-thick slices. The sections were prepared and stained with hematoxylin and eosin (H&E) and Masson trichrome stain. The histological slides were evaluated by a blind independent pathologist, using light microscopy (Olympus BX51; Olympus, Tokyo, Japan) at 10x and 40x magnifications. The number of lymphocytes, eosinophils, neutrophils, and osteoclasts in different samples were measured and compared. In addition, for histomorphometric analysis, the number of empty blood vessels and empty lacunae were analyzed and calculated by software Image-Pro Plus® V.6 (Media Cybernetics, Inc., Silver Spring, USA). In addition, the percentage of the live and necrotic bones were evaluated in different groups. The pathologist was not aware of the grouping of the slides during microscopic examination and the information about these cases was provided to the examiner in coded form.

### Statistical analysis

Descriptive statistics were calculated. Kruskal-Wallis test was used to compare the groups in terms of quantitative histomorphometric parameters. The level of significance was set at 0.05.

## Results

### Qualitative assessments

Microscopic examinations are presented in Figs. 2, 3, 4, 5, 6, 7, 8, 9, 10, 11, 12, 13, 14, 15, 16, 17, 18, 19, 20, 21, 22, 23, 24, and 25.

**Fig. 2:**
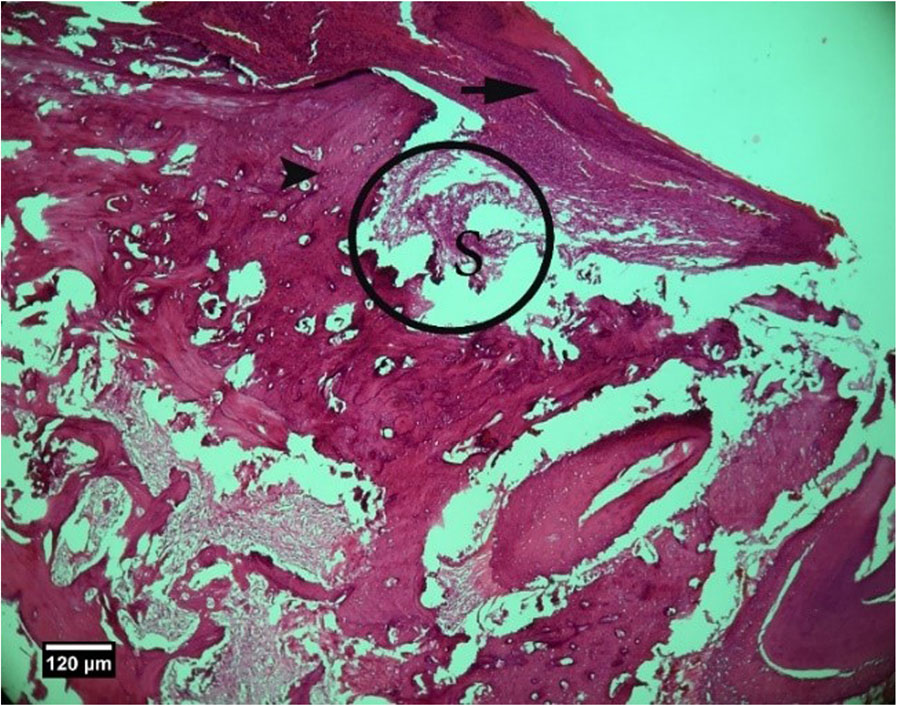
Alveolar bone cross-section is seen in the control group without treatment 2 weeks after tooth extraction, dental socket (S), alveolar bone (arrowhead), and enlarged epithelium (arrow) (H&E)

**Fig. 3:**
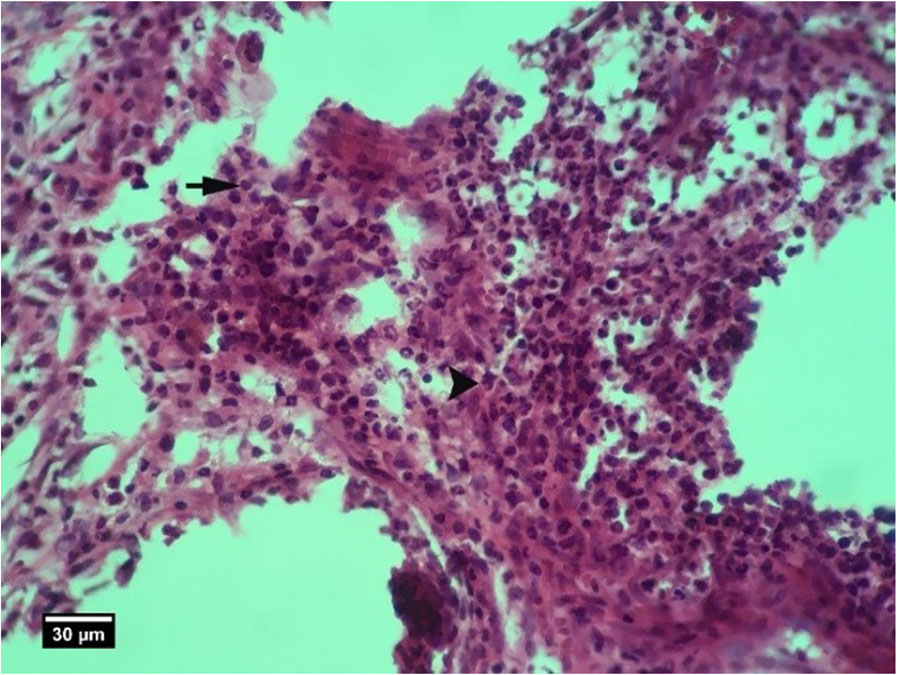
Socket section of the control group without treatment 2 weeks after tooth extraction, severe infiltration of neutrophil inflammatory cells (arrow tip) and lymphocytes (arrow) are seen inside the socket (H&E)

**Fig. 4:**
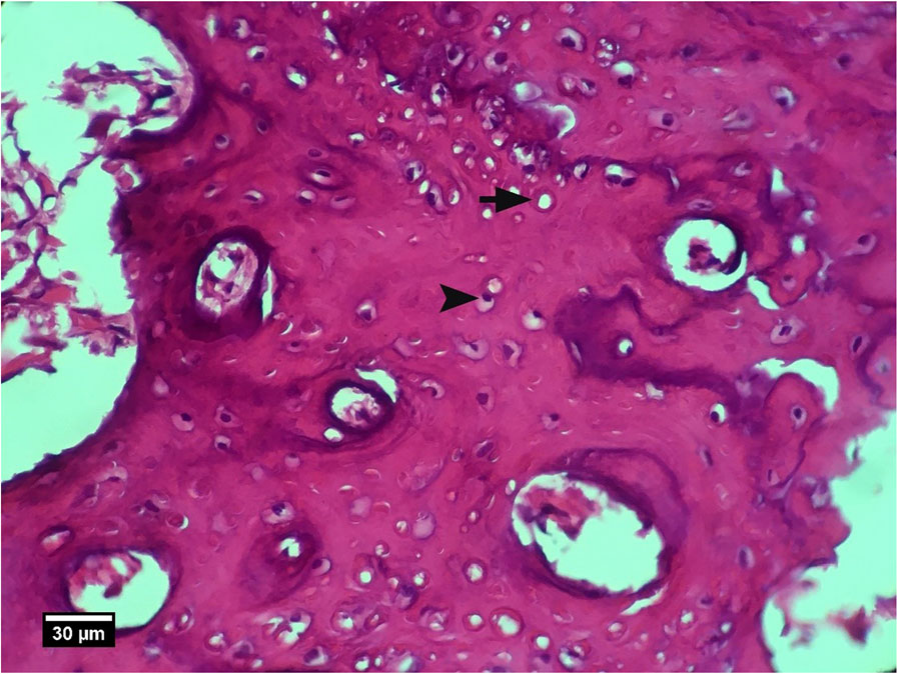
Alveolar bone cross-section in the control group without treatment 2 weeks after tooth extraction, a significant number of empty lacunae (arrow) and lacunae with pyknotic nuclei (arrow tip) are seen (H&E)

**Fig. 5:**
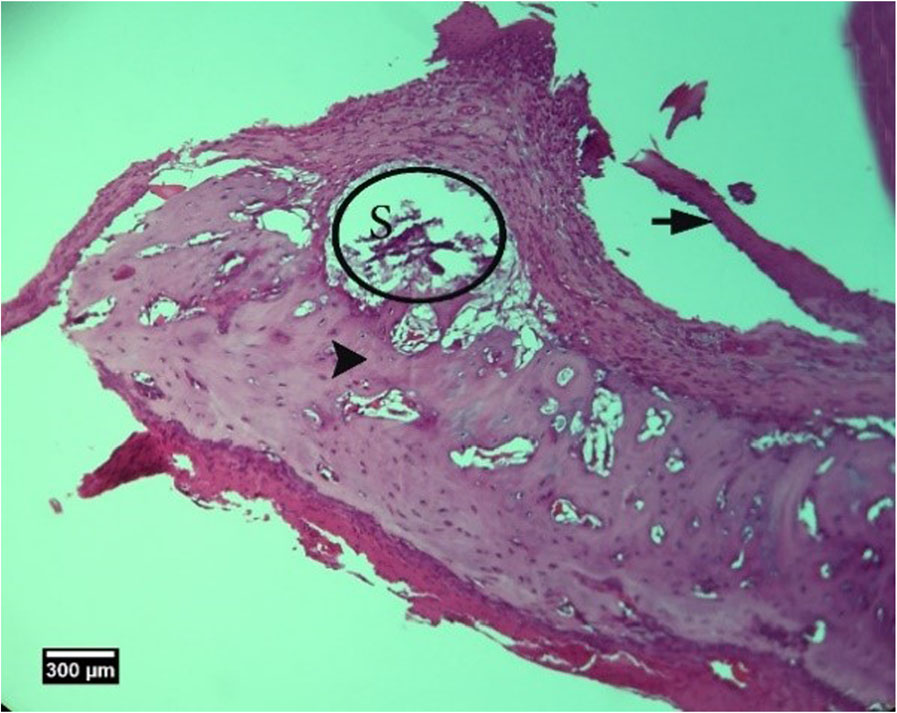
Alveolar bone cross-section is seen in group HA 2 weeks after tooth extraction, dental socket (S), alveolar bone (arrow tip), and enlarged epithelium (arrow) (H&E)

**Fig. 6:**
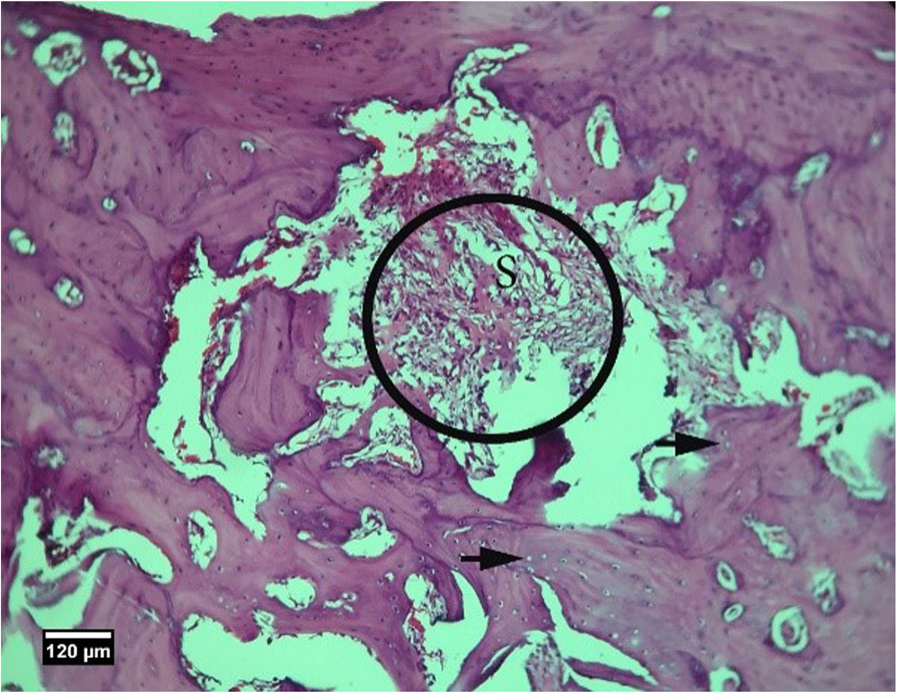
Alveolar bone cross-section. In HA group, 2 weeks after tooth extraction, dental socket (S) and a small number of empty lacunae (arrow) are seen (H&E)

**Fig. 7:**
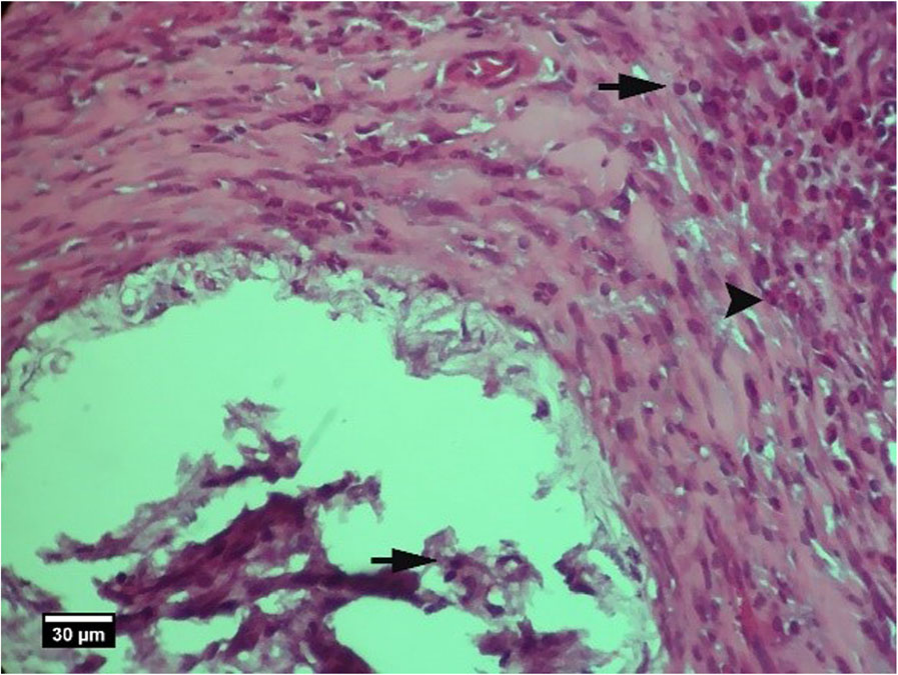
Section of soft tissue around the socket. In the HA group, 2 weeks after tooth extraction, infiltration of eosinophil (arrow tip) and lymphocyte (arrow) inflammatory cells are seen inside the socket (H&E)

**Fig. 8:**
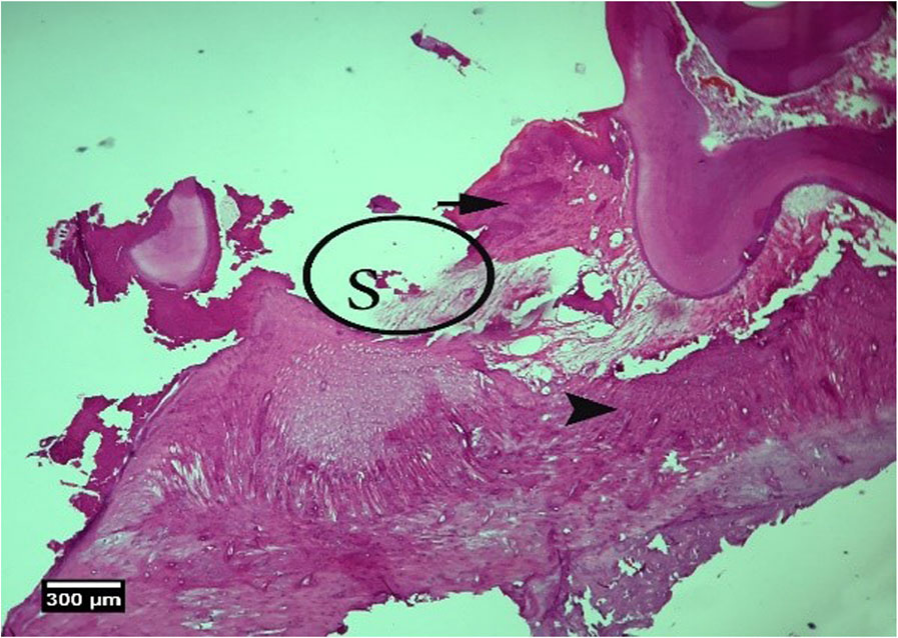
Alveolar bone cross-section is seen in ACS group 2 weeks after tooth extraction, dental socket (S), alveolar bone (arrow tip), and enlarged epithelium (arrow) (H&E)

**Fig. 9:**
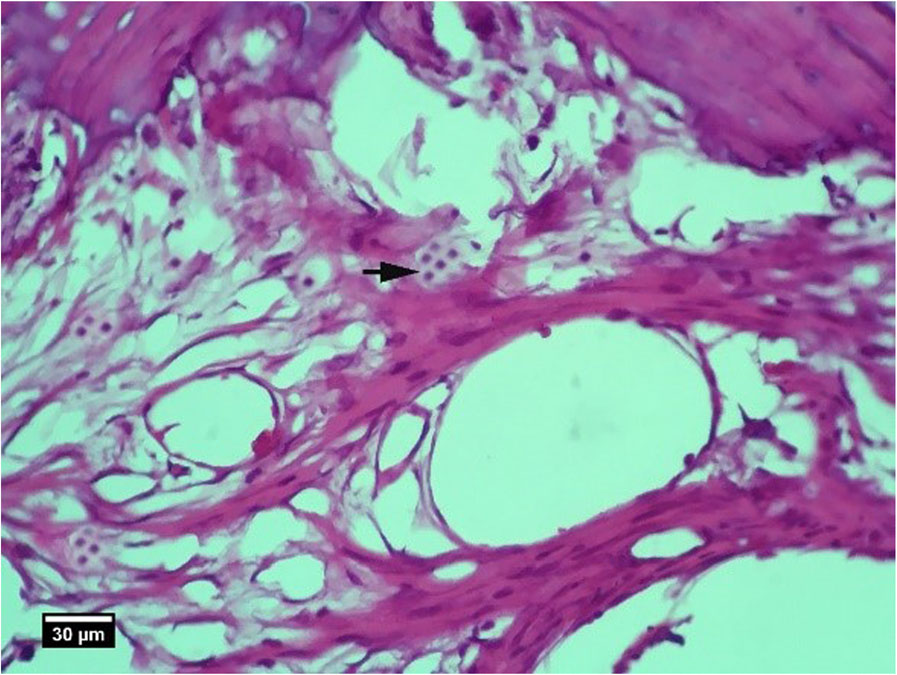
Socket section of the ACS group 2 weeks after tooth extraction, infiltration of eosinophil inflammatory cells (arrow tip), and lymphocytes (arrow) is seen inside the socket (H&E)

**Fig. 10:**
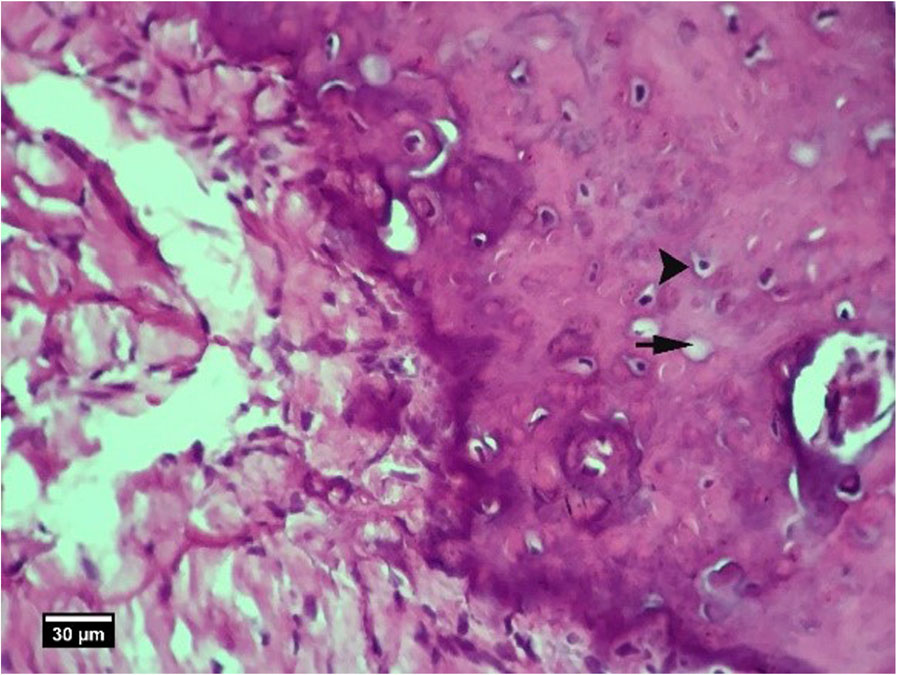
Alveolar bone cross-section in ACS group 2 weeks after tooth extraction, a number of empty lacunae (arrow) and lacunae with pyknotic nuclei (arrow tip) are seen (H&E)

**Fig. 11:**
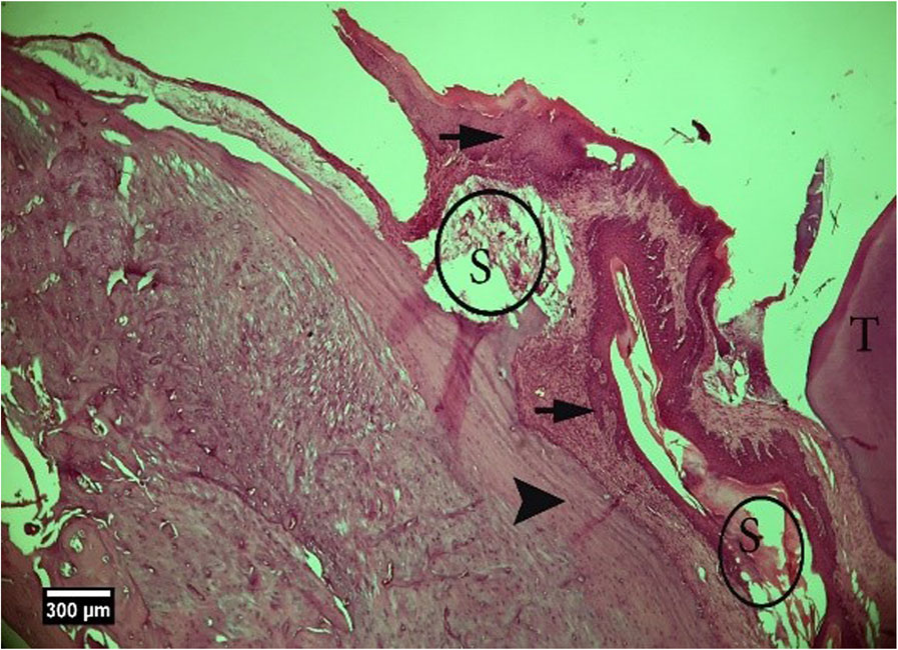
Alveolar bone cross-section in ACS + HA group 2weeks after tooth extraction, dental socket (S), alveolar bone (arrow tip), and enlarged epithelium (arrow) and cross-section of an adjacent tooth (T) are seen (H&E))

**Fig. 12:**
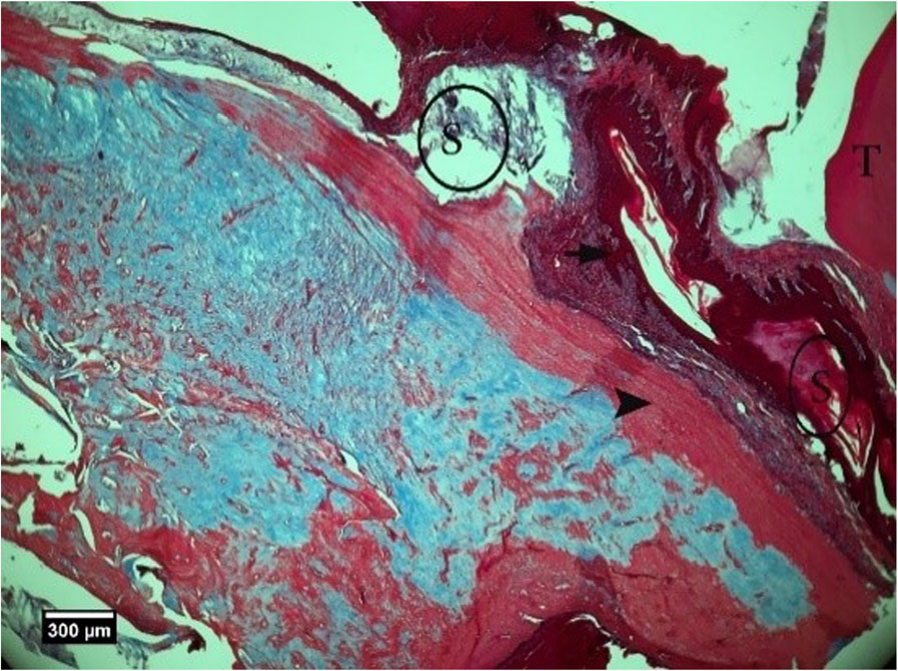
Alveolar bone cross-section in ACS + HA group 2 weeks after tooth extraction, dental socket (S), alveolar bone (arrow tip), and enlarged epithelium (arrow) and cross-section of an adjacent tooth (T) are seen (Trichrome)

**Fig. 13:**
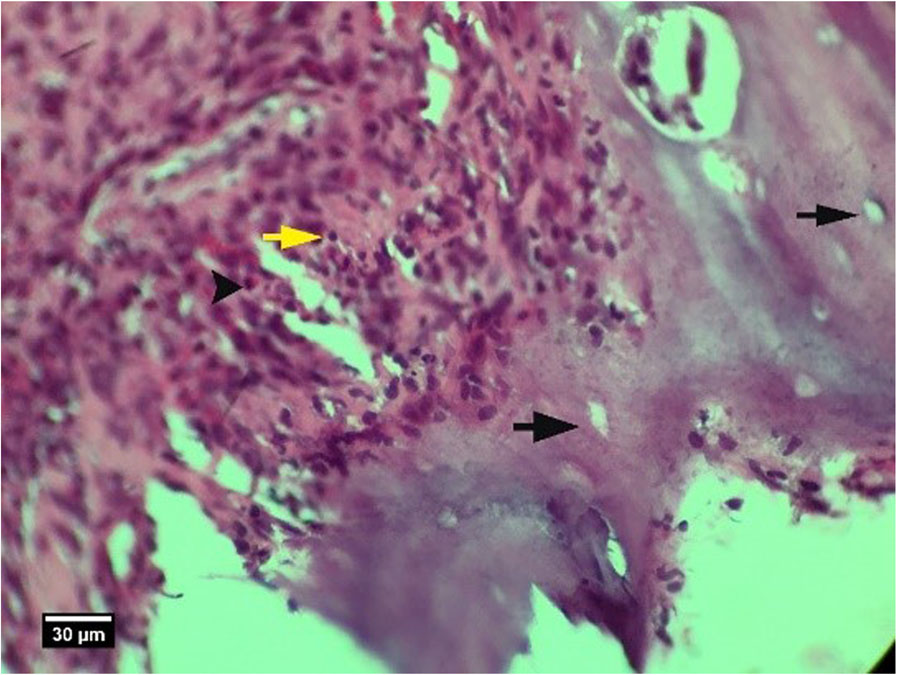
Alveolar bone cross-section and soft tissue around the socket in the HA + ACS group 2 weeks after tooth extraction, a number of empty lacunae (arrow), proliferation of eosinophil inflammatory cells (arrowhead), and lymphocytes (yellow arrow) are seen inside the socket (H&E)

**Fig. 14:**
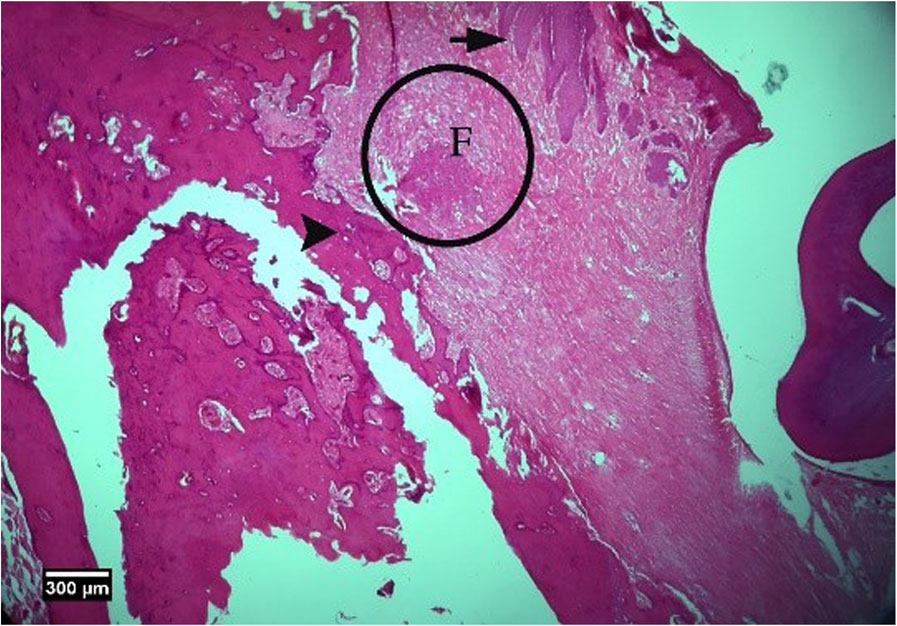
Alveolar bone cross-section in the control group without treatment 8 weeks after tooth extraction, restorative tissue is seen in the dentate gyrus (F), alveolar bone (arrow tip), and enlarged epithelium (arrow) (H&E)

**Fig. 15:**
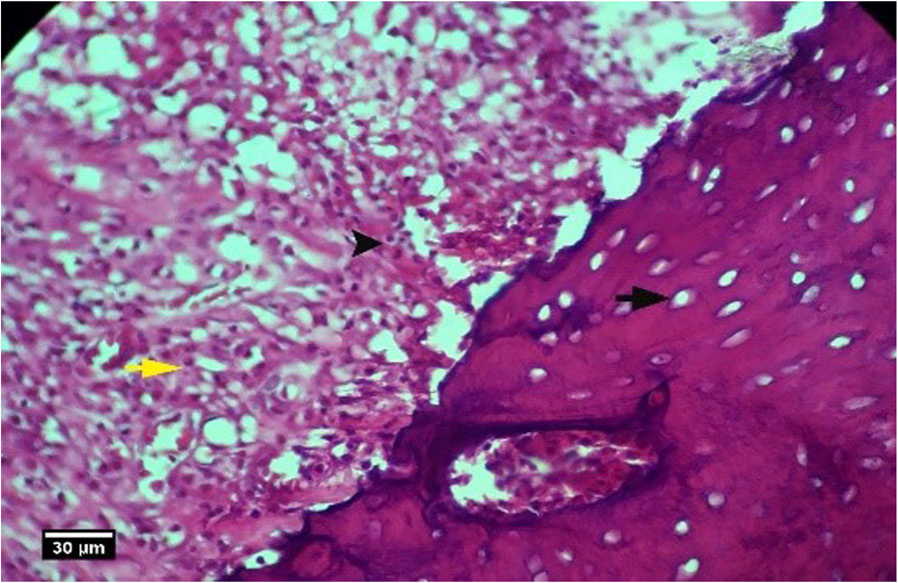
Socket section of the control group without treatment 8 weeks after tooth extraction, severe infiltration of eosinophil (yellow arrow), and lymphocyte (arrow tip) inflammatory cells is seen inside the socket and a large number of empty lacunae (arrow) in the alveolar bone (H&E)

**Fig. 16:**
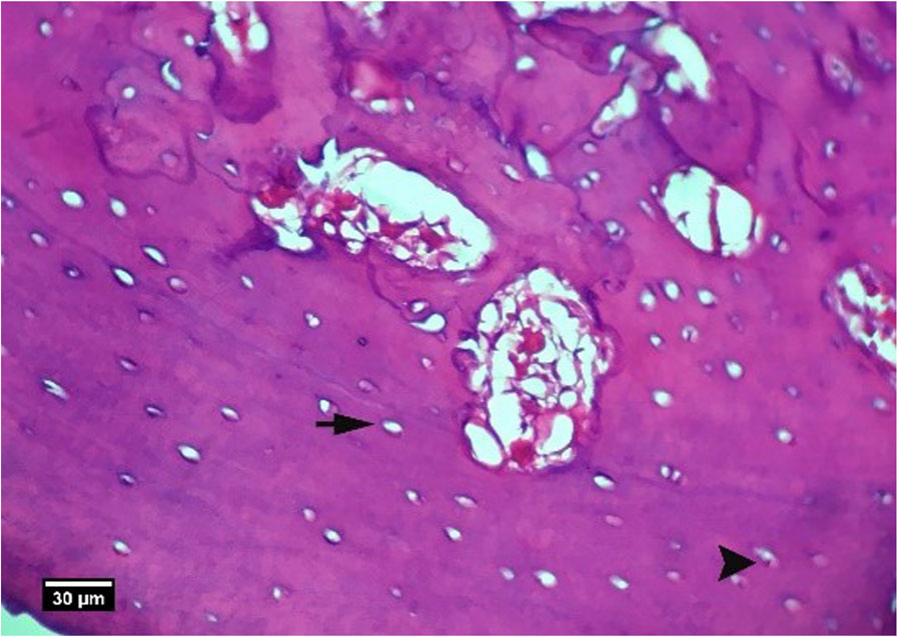
Alveolar bone section in the control group without treatment 2 weeks after tooth extraction: a large number of empty lacunae (arrow) and lacunae with pyknotic nuclei (arrow tip) are seen (H&E)

**Fig. 17:**
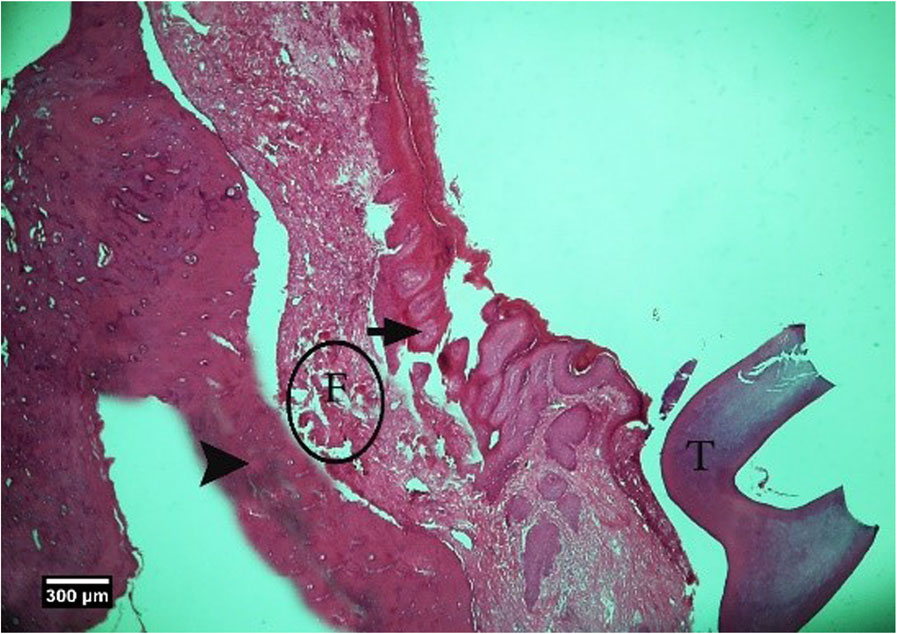
Alveolar bone cross-section in HA group 8 weeks after tooth extraction, restorative tissue in the dental cavity (F), alveolar bone (arrow tip), and enlarged epithelium (arrow) and part of adjacent tooth (T) are seen (H&E)

**Fig. 18:**
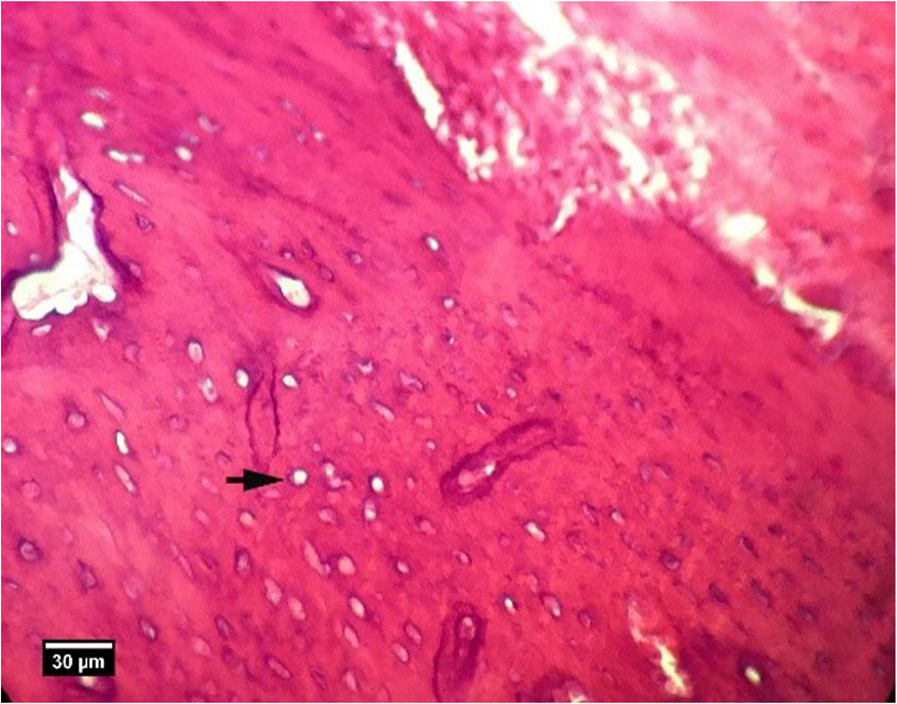
Alveolar bone section. In group HA, 8 weeks after tooth extraction, some empty lacunae (arrow) are seen (H&E)

**Fig. 19:**
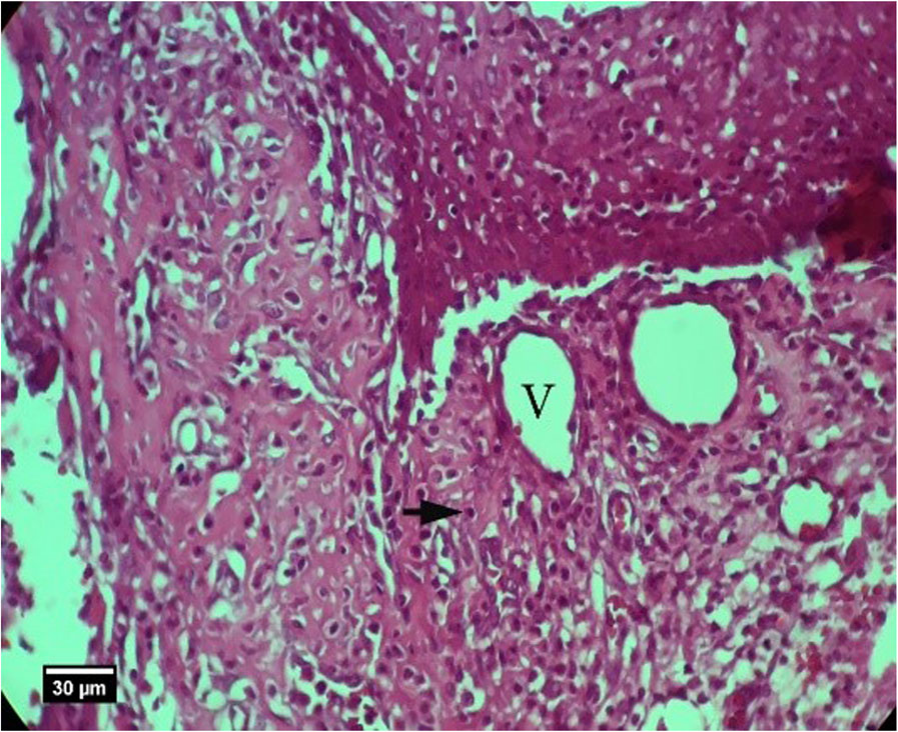
Soft tissue section around the silent in the HA group, 8 weeks after tooth extraction: infiltration of inflammatory cells of lymphocytes (arrow) and blood vessels (V) is seen inside the socket (H&E)

**Fig. 20:**
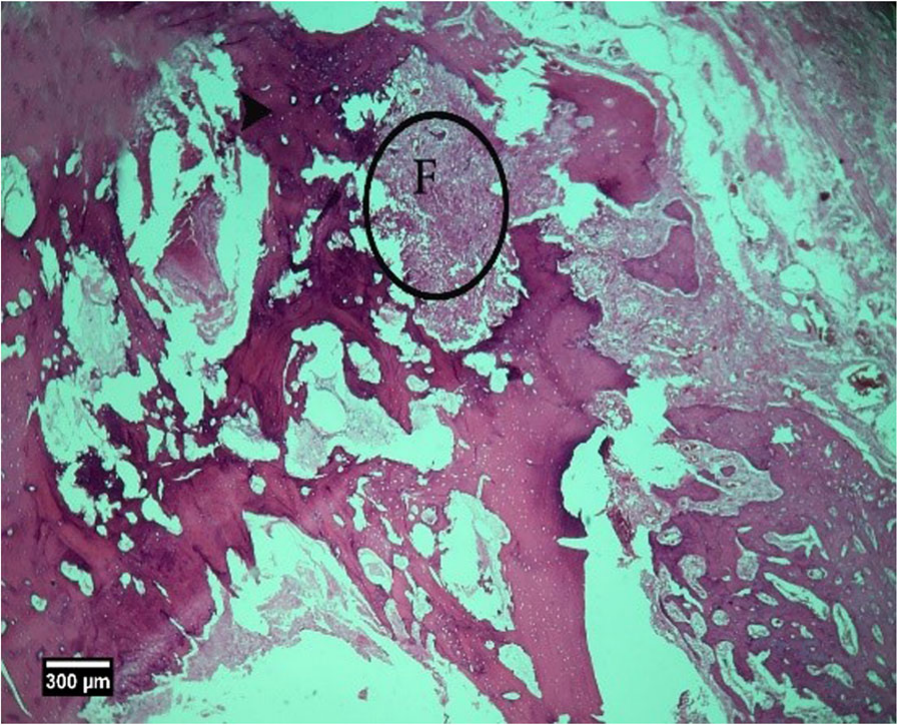
Alveolar bone cross-section in ACS group, 8 weeks after tooth extraction: restorative tissue is seen in the dental socket (F) and alveolar bone (arrow tip) (H&E)

**Fig. 21:**
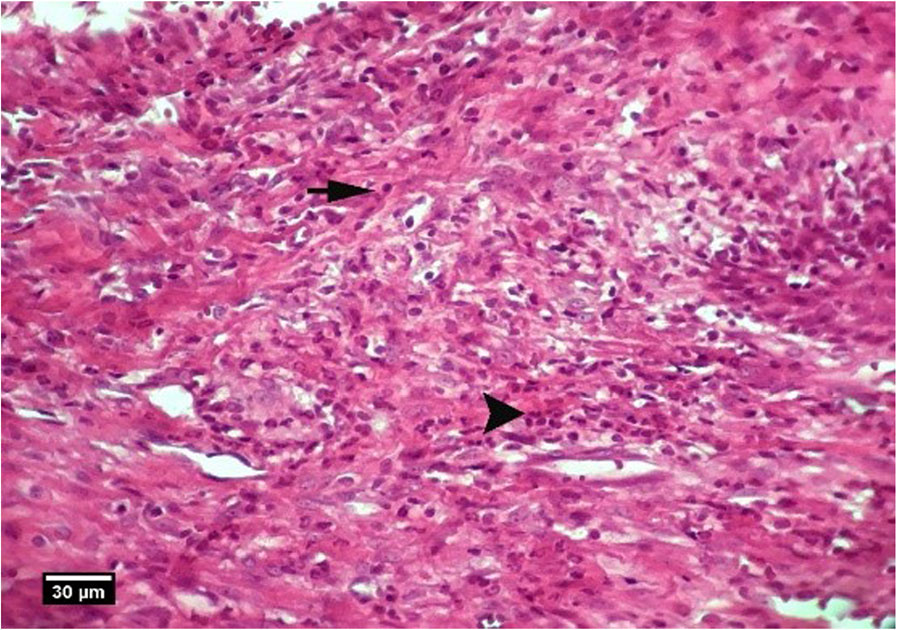
Socket section of ACS group, 8 weeks after tooth extraction: penetration of neutrophil (arrow tip) and lymphocyte (arrow) inflammatory cells is seen inside the socket (H&E)

**Fig. 22:**
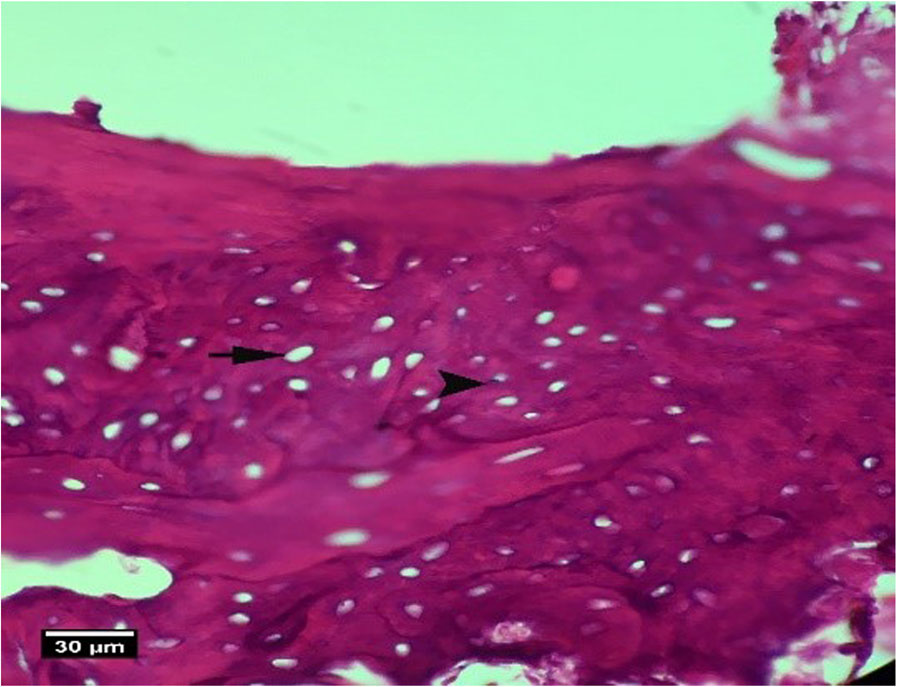
Alveolar bone cross-section in ACS group, 8 weeks after tooth extraction: a significant number of empty lacunae (arrow) and lacunae with pyknotic nuclei (arrow tip) are seen (H&E)

**Fig. 23:**
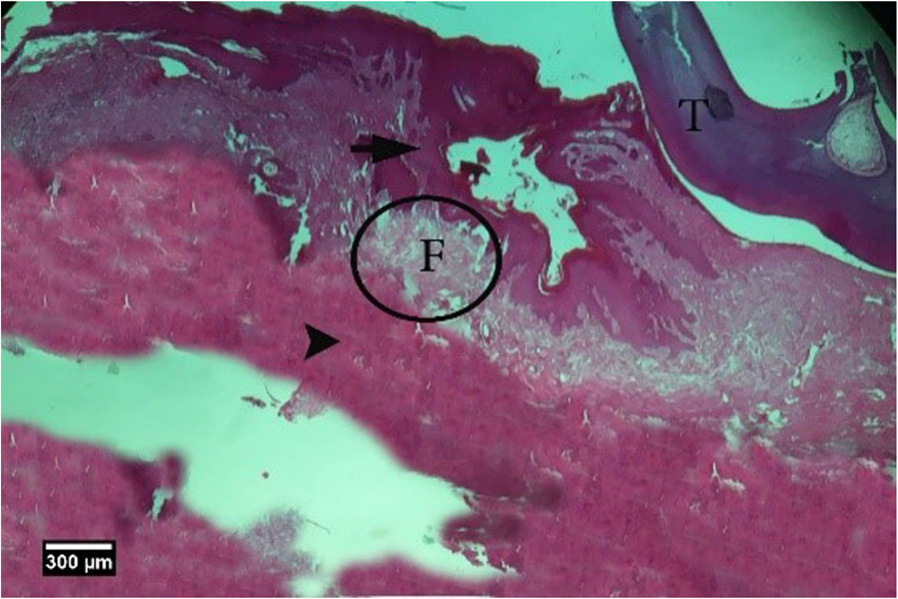
Alveolar bone cross-section in ACS + HA group, 8 weeks after tooth extraction: restorative tissue in the dental cavity (F), alveolar bone (arrow tip), and enlarged epithelium (arrow) and cross-section of tooth

**Fig. 24:**
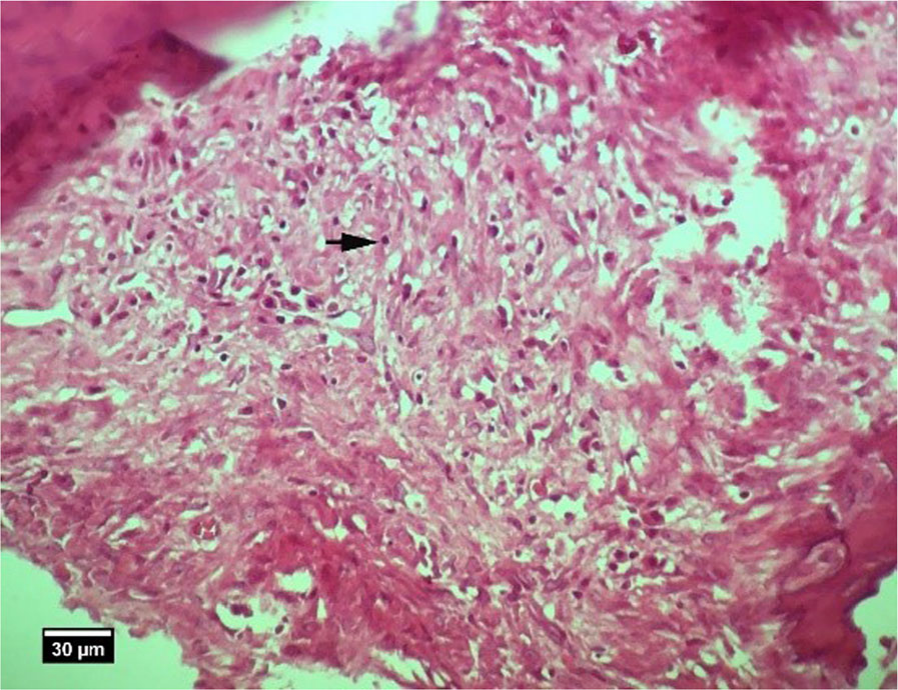
Alveolar bone cross-section and soft tissue around the socket. In the HA + ACS group, 8 weeks after tooth extraction: slight infiltration of inflammatory lymphocyte cells (arrow) is seen inside the socket (H&E)

**Fig. 25:**
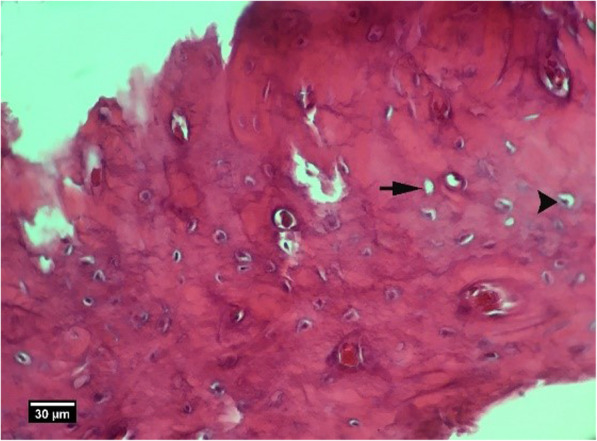
Alveolar bone cross-section in the HA + ACS group, 8 weeks after tooth extraction: a small number of empty lacunae (arrow) and lacunae with pyknotic nuclei (arrow tip) are seen (H&E)

### Quantitative assessments

Six rats died after surgery (2 in the HA group, 2 in the ACS group, and 2 in the control group) and statistical analyses were done on 34 rats. The average number of the blood vessels by groups and separately for the second and eighth weeks is presented in Table 1 and shows that in the second week, the maximum average number of the blood vessels in the third group (ACS) is 6.0 ± 1.41 with a coefficient of variation of 23, and the lowest, i.e., the second group (ACS + HA), was 2.6 ± 0.54 with a coefficient of variation of 20; the test showed that the difference was statistically significant (*P* < 0.05). In the eighth week, the highest average number of the blood vessels in the second group (ACS + HA) was 4.2 ± 1.3 with a coefficient of variation of 30 and the lowest blood vessel rate was for the control group (3 ± 0.7 with a coefficient of variation of 23). The test showed that this difference was not statistically significant (*P* = 0.4).

**Table 1:** The average number of histopathologic components in different groups

Variable	Vessels		Osteoclasts		Empty lacunae	
	Second week	Eighth week	Second week	Eighth week	Second week	Eighth week
**HA**	4.75 ± 0.96	3.5 ± 1.73	1.25 ± 0.5	0.75 ± 0.5	1.5 ± 1.29	1.25 ± 1.25
**HA + ACS**	2.6 ± 0.54	4.2 ± 1.3	1.6 ± 0.55	0.6 ± 0.89	4.6 ± 2.07	0.8 ± 0.83
**ACS**	6.0 ± 1.41	3.5 ± 1.29	1 ± 0.81	1.25 ± 1.25	3.25 ± 1.25	21.5 ± 7.59
**Control**	4.25 ± 1.89	3.0 ± 0.7	1.5 ± 0.57	3.25 ± 2.75	11.75 ± 3.09	22.5 ± 9.25
***P*** **value**	< 0.05	0.4	0.7	< 0.05	< 0.05	< 0.05

The average number of osteoclasts was examined in terms of frequency and presented in four groups in Table 1 which shows that in the second week, the highest rate was in the second group (ACS + HA) as 1.6 ± 0.55 with a coefficient of variation of 34 and the lowest was in the third group (ACS) as 1 ± 0.81 with a coefficient of variation of 81; this difference was not significant (*P* = 0.7). In the eighth week, the highest number of osteoclasts belonged to the control group at 3.25 ± 2.75 with a coefficient of variation of 85% and the lowest was the second group (ACS + HA) at 0.62 ± 0.86 with a coefficient of variation of 148%. This difference was statistically significant (*P* < 0.05).

In the second week, the highest average number of empty lacunae belonged to the control group at 11.75 ± 3.09 and the lowest number was in the first group (HA) as 1.5 ± 1.29. This difference was statistically significant (*P* < 0.05). At the 8th week follow-up, the highest number of empty lacunae in the control group was 22.5 ± 9.25 and the lowest number was in the second group (ACS + HA) (0.8 ± 0.83), which was statistically significant (*P* < 0.05).

The number of eosinophils in soft tissue is presented in Table 2 and shows that in the second week the minimum number in the control group was 0.75 ± 0.95 with a coefficient of variation of 126. The greatest number belonged to the first group (HA) which was 7.75 ± 2.75 with a coefficient of variation of 35% and this difference was statistically significant (*P* < 0.05). In the eighth week, the highest number in the control group was 6.5 ± 5.06 with a coefficient of variation of 77.8% and the lowest number was in the second group (ACS + HA) at 2.2 ± 2.28 with a coefficient of variation of 103 and this was statistically significant (*P* < 0.05, Table 2).

**Table 2:** The average number of immunological components in different groups

Variable	Eosinophils		Lymphocytes		Neutrophils	
	Second week	Eighth week	Second week	Eighth week	Second week	Eighth week
**HA**	7.75 ± 2.75	2.5 ± 3	8.25 ± 2.98	18.25 ± 3.30	39.5 ± 5.68	2 ± 1.41
**HA + ACS**	3.2 ± 0.83	2.2 ± 2.28	15.6 ± 2.79	12.2 ± 1.78	50.4 ± 11.63	1.4 ± 1.34
**ACS**	3.25 ± 5.25	4.25 ± 2.96	5.5 ± 1.73	10 ± 1.63	97.5 ± 114	20.75 ± 2.87
**Control**	0.75 ± 0.95	6.5 ± 5.06	15.25 ± 3.77	8.25 ± 1.5	19.75 ± 22.42	41.25 ± 12.4
***P*** **value**	< 0.05	< 0.05	< 0.01	< 0.02	< 0.01	< 0.01

In the second week, the highest lymphocyte count in the soft tissue belonged to the second group (ACS + HA) at 15.6 ± 2.79 with a coefficient of variation of 17.9% and the lowest belonged to the third group (ACS) at 5.5 ± 1.73 with a coefficient of 31.5% (*P* < 0.01, Table 2). In the eighth week of follow-up, the highest rate was related to the first group (HA) at 18.25 ± 3.3 with a coefficient of variation of 18% and the lowest rate belonged to the control group at 8.25 ± 1.5 with a coefficient of variation of 18%, which was statistically significant (*P* < 0.02, Table 2).

The average number of neutrophils in soft tissue shows that in the second week, the highest neutrophil count belonged to the third group (ACS) at 97.5 ± 114 with a coefficient of variation of 116% and the lowest belonged to the control group at 22.75 ± 19.4 with a coefficient of variation of 113%. This difference was statistically significant (*P* <0.01, Table 2). In the eighth week, the highest neutrophil count was 41.25 ± 5.12 in the control group with a coefficient of variation of 12.4% and the lowest was related to the second group (ACS + HA) at 1.4 ± 1.34 with a coefficient of variation of 95.7% (*P* < 0.01, Table 2).

The percent of the necrotic bone was measured (Table 3) which shows that in the second week, the highest percentage of the necrotic bone belonged to the first group (HA) at 3.0 ± 4.76 with a coefficient of variation of 158% and the lowest percentage belonged to the third group (ACS, 0.93 ± 0.94) with a coefficient of variation of 101%; the overall difference was not significant (*P* = 0.6, Table 3). In the eighth week, the highest percentage of bone necrosis was observed in the control group at 30 ± 9.8 with a coefficient of variation of 32% and the lowest belonged to the second group (ACS + HA) at 2.02 ± 4.46 with a coefficient of variation of 220%, which was statistically significant (*P* < 0.04).

**Table 3:** The average percentage of bone structures in different groups

Variable	Necrotic bone		Vital bone	
	Second week	Eighth week	Second week	Eighth week
**HA**	3 ± 4.76	4.125 ± 6.69	97 ± 4.76	95.88 ± 6.68
**HA + ACS**	2.4 ± 2.04	2.02 ± 4.46	97.6 ± 2.04	97.98 ± 4.46
**ACS**	0.93 ± 0.94	13.33 ± 16.12	99.07 ± 0.94	86.68 ± 16.13
**Control**	2.25 ± 3.30	30.05 ± 9.85	97.75 ± 3.30	69.95 ± 9.85
***P*** **value**	0.6	< 0.04	0.6	< 0.04

Table 3 shows percentage of the vital bone; in the second week, the highest percentage belonged to the third group (ACS) with 99.32 ± 0.94 with a coefficient of variation of 0.94% and the lowest was observed in the first group (HA) which was 97% with a coefficient of variation of 4.9%; the overall difference was not significant (*P* = 0.6). In the eighth week, the highest percentage was seen in the second group (ACS + HA, 97.98 ± 4.46) and the lowest rate was related to the control group (69.95 ± 9.85), which was statistically significant (*P* < 0.04, Table 3).

## Discussion

Since there is no similar study, we are limited to discussing our results in light of more general topics. In the second week of the present study, the number of vessels in the ACS group was higher than in the HA + ACS group, which can be related to the property of ACS as a substance for keeping blood clots in the area and the presence of more mesenchymal cells in it. The number of vessels in the eighth week reached similar values in all groups. Although the number of osteoclasts was not different between different groups in the second week, it reached a significantly higher number in the control group in the eighth week, indicating a higher rate of bone resorption in the control group. The number of empty lacunae shows a decrease in the repair process in the control group, which continued in the eighth week of the study and resulted in an increase in empty lacunae in the control group. This implied reduced BRONJ signs. The number of eosinophils in the HA group was higher than the control group and other groups in the second week; HA may have caused an allergic reaction due to the low molecular weight, and the immune response may be related to it. In the HA + ACS group and ACS group however, eosinophils were not as increased, which might be due to the more controlled release of HA from ACS as well as the lack of HA. However, the number of eosinophils grew higher in the control and ACS groups in the eighth week. The number of lymphocytes was greater in both the control and HA + ACS groups in the second week, and in the HA group in the eighth week. The number of neutrophils in the ACS group was higher than the rest in the second week, which can be due to a higher rate of post-surgical infection. However, in the eighth week, the control group had the highest number of eosinophils. Due to the restorative properties of hyaluronic acid and prevention of bone necrosis, we have a decreasing trend in the number of neutrophils, which indicates the absence of active infection in the tissue. Although the percentage of the necrotic bone was not significantly different in the second week, it became higher in control group followed by ACS group; in the two groups containing HA, it remained low in the eighth week. This indicates the role of HA in bone regeneration.

This study showed for the first time that HA (with or without ACS) can improve bone healing in BRONJ. This is line with previous studies that had shown enhancing effects of HA in bone growth [24–26] and mineralization [27]. For instance, Schulz et al. [28] implanted the maxillae of miniature pigs with titanium dental implants coated with HA on their surfaces. Their results indicated that in the early healing period, HA improves bone formation at the bone-implant junction [28]. Nguyen and Lee [27] developed a scaffold of HA hydrogel loaded into a biphasic calcium phosphate ceramic. Their new bone substitute showed a high rate of collagen mineralization and fast new bone formation [27]. Krause et al. [29] evaluated the efficacy of a novel paste comprising HA. This bone substitute indicated an early bone formation [29]. Another study [30] as well confirmed the results of earlier studies concluding that the proper effect of HA on bone regeneration is visible at an early phase of healing because HA is an element of the extracellular matrix which acts as a scaffold for mesenchymal cell migration [31], allowing them to differentiate, proliferate [32, 33], and migrate [34] which in turn induced growth of osteoblasts and osteocytes [30].

Physical properties of HA can largely influence the regenerative potential of HA [30]. The reticulated HA might show a superior regenerative ability in comparison with linear HA [35]. Besides, molecular weight and particle size as well considerably influence the HA function [13, 36]. HAs with high molecular weights might increase the mRNA expressions of RUNX-2, ALP, and OCN [37], inducing bone formation [30]. The degree of crosslinking along with molecular weight determine the viscosity of HA [30]. Despite the frequent use of hydroxyapatite granules for repairing bone defects, their dried forms are difficult to handle in the surgical room because of lacking cohesion and low weight [35]. Therefore, adding such particles into a hydrogel could be a probable method for improving both ease of handling and regenerative efficiency [30, 38, 39].

ACS can as well play a role. For optimal effect, agents such as rhBMP-2 should be carried by suitable scaffolds to stabilize their release into the lesion. These scaffolds may be made of the demineralized bone matrix, tricalcium phosphate, hydroxyapatite, and ACS. The latter has been shown to prolong the release of rhBMP-2 [23, 40, 41]. It has been shown that collagen enriched with growth factors can induce osteogenesis or enhance cellular colonization and increase cell adhesion, viability, and proliferation [21]. Collagen is the most commonly used carrier for the delivery of substances such as rhBMPs, since it is the most copious non-mineral skeletal element [42] and can enhance bone formation [43]. Nevertheless, collagen as a carrier might have drawbacks [21, 42]. It is mechanically weak as a scaffold: unwanted release can occur, and its biodegradation is difficult to control and unpredictable; this might result in kinetic release of the collagen [22, 42].

This preliminary study was limited by some factors. The original sample size was not determined based on power calculations, and the lost rats were not replaced by new ones to maintain the original size. Still, the remaining sample size sufficed to provide many significant findings. A split-mouth design could increase the power; however, this was not possible with more than 2 groups; besides, the trauma of bilateral surgery could pose a death risk to many rats. Finally, animal studies might not be generalized to humans. Nevertheless, since there was no study on HLA with or without ACS, our preliminary research needed to confirm its results first in animals. Also histopathological assessments were impossible in humans.

## Conclusion

Within the limitations of this preliminary histological animal study, it might be concluded that HA + ACS followed by HA can be proper methods for filling the extraction socket, instead of ACS alone or leaving the socket empty. HA + ACS might be favored over HA alone, not only because of the superior results, but also because of improved sustenance and handling of the HA gel. Future researches with larger samples and possibly with human subjects are recommended to examine the effectiveness of HA with or without ACS in treating or preventing BRONJ.

## Data Availability

The data are not available.
